# Transitioning to Working from Home Due to the COVID-19 Pandemic Significantly Increased Sedentary Behavior and Decreased Physical Activity: A Meta-Analysis

**DOI:** 10.3390/ijerph21070851

**Published:** 2024-06-28

**Authors:** Nicole Chaudhary, Megan Jones, Sean P. M. Rice, Laura Zeigen, Saurabh Suhas Thosar

**Affiliations:** 1 Institute of Occupational Health Sciences, Oregon Health & Science University (OHSU), 3181 SW Sam Jackson Park Rd, Portland, OR 97239, USA; burkeni@ohsu.edu (N.C.); jonmegan@ohsu.edu (M.J.); ricese@ohsu.edu (S.P.M.R.); 2 School of Public Health, Oregon Health & Science University-Portland State University (OHSU-PSU), Portland, OR 97239, USA; 3 Oregon Health & Science University Library, Oregon Health & Science University (OHSU), Portland, OR 97239, USA; zeigenl@ohsu.edu; 4 School of Nursing, Oregon Health & Science University (OHSU), Portland, OR 97239, USA; 5 Knight Cardiovascular Institute, School of Medicine, Oregon Health & Science University (OHSU), Portland, OR 97239, USA

**Keywords:** COVID-19, work from home, physical activity, remote work, sedentary behavior, chronic disease risk, future of work initiative

## Abstract

At the start of the COVID-19 pandemic, many workplaces transitioned to remote work, which altered lifestyle behaviors. We conducted a meta-analysis to understand if the transition to working from home due to the pandemic affected workers’ physical activity and sedentary behavior worldwide. We reviewed articles published between November 2019 and May 2022. Of an initial 3485 articles, a total of 17 were included, 15 of 17 were included for their physical activity (PA) comparisons (*n* = 36,650), and 12 of 17 (*n* = 57,254) were included for their sedentary behavior (SB) comparisons (10 studies have data for both PA and SB). This work is registered through PROSPERO (CRD42022356000). Working from home resulted in a significant decrease in PA (Hedge’s *g* = −0.29, 95% CI [−0.41, −0.18]) and an increase in SB (Hedge’s *g* = +0.36, 95% CI [0.20, 0.52]). Working from home impaired preventative activity behaviors, and these results are relevant to worker health as the future of work evolves beyond the pandemic.

## 1. Introduction

In March 2020, the World Health Organization (WHO) declared COVID-19 a pandemic, necessitating overwhelming changes in daily life across the globe. Several changes and adaptations were made to slow the spread of the virus and help contain SARS-CoV-2 [1]. Along with a worldwide shutdown of in-person events (e.g., schools, gatherings, entertainment, and travel), the nature of work was fundamentally altered. Several workers whose jobs did not require in-person presence in office buildings were abruptly asked to work from home (WFH) to fulfill their job duties. This change in work practices has persisted, and lifestyle behaviors have been altered, including physical activity (PA) and sedentary behavior (SB) [2,3,4,5,6,7,8,9,10].

Physical activity is a major preventative behavior associated with reduced risks of chronic and lifestyle diseases, such as cardiovascular diseases, obesity, and diabetes [11]. On the other hand, sedentary behavior, defined as prolonged periods spent in a sitting, reclining, or lying down posture or walking with low energy expenditure (<1.5 METS—metabolic equivalent units), is associated with poor health outcomes and risk for lifestyle diseases [12,13,14]. Varying guidelines have been suggested regarding time spent performing physical activities. The Centers for Disease Control and Prevention (CDC) recommend that adults perform 150 min of moderate-intensity aerobic physical activity or 75 min of vigorous-intensity physical activity each week to maintain overall health [11]. Similarly, the World Health Organization (WHO) recommends limiting sedentary behavior, and additional recommendations include desk-based workers starting with 2 h per day of standing and light activity (e.g., walking) during work hours [12,15]. There are several domains of physical activity, including occupational pursuits, leisure activities, domestic work, and transportation [16]. Assuming the average working adult works for approximately 8 h each day, these people spend over one-third of their waking hours in occupational pursuits. Therefore, in these adults, work-related activities can contribute significantly to their weekly volume of physical activity, sedentary behavior, and consequent overall health. Furthermore, work-related activities like commuting can contribute to these daily behaviors. For instance, active commuting, such as biking to work, increases physical activity, whereas driving to work is a primarily sedentary form of transportation. Thus, work and work-related activities are likely to majorly impact the overall physical activity levels and sedentary behaviors in employed adults. An abrupt shift to working from home or hybrid work can significantly affect a worker’s PA and SB habits and influence their overall health.

As shown in previous literature, prior to the pandemic, workers may have spent as much as 77% of their workday being sedentary [17]. Furthermore, working from home affected “pain, self-reported health, safety, well-being, stress, depression, fatigue, quality of life, strain and happiness” and could be influenced by workplace supports [18]. It has been reported that white-collar workers (e.g., desk workers) have lower levels of PA and higher SB compared to blue-collar workers [19]. Considering that work can influence physical activity and sedentary behavior, we sought to determine the effect of a worldwide shift to working from home as a result of the pandemic.

Several researchers across the world have investigated these COVID-19 pandemic-related changes in activity behaviors, with mixed results [2,3,4,5,20,21]. This meta-analysis aimed to summarize the impact that COVID-19-enforced transitions to work-from-home had on physical activity and sedentary behavior. We sought to specifically identify how the abrupt change in work practices due to the pandemic affected physical activity (PA) and sedentary behavior (SB). We hypothesized that the sudden transition to working from home would significantly increase sedentary behaviors and significantly decrease physical activities in the affected working adults.

## 2. Materials and Methods

### 2.1. Experimental Approach

This review was registered with PROSPERO (CRD42022356000). The earliest cases of illness in the COVID-19 pandemic were most likely detected in November 2019 [22], and to ensure all potential literature were captured within the search, the search date range was November 2019 through May 2022. We included articles if they: (a) were original research, (b) explored a change in physical activity or sedentary behavior due to working from home during the COVID-19 pandemic, (c) included data collected during the pandemic and were published between November 2019 and May 2022, (d) included people working from home as a result of the pandemic, (e) measured physical activity/sedentary behaviors using subjective or objective measurements, and (f) were written in English. We excluded studies where a majority of the sample included students or studies that did not separate the working population from the sample (e.g., children, adolescents, or people who were not working/were retired) because these populations did not reflect the average working population.

Inclusion in the meta-analysis required the studies to have either compared work-from-home (WFH) to non-WFH employees during the pandemic (between subjects) or studies in which a majority of the workers changed from on-site to remote work as a result of the pandemic (within subjects). Additionally, we only included studies in which the authors reported comparable effect sizes (i.e., those that could be converted into one another, such as Cohen’s d values, odds ratios, and/or correlations) or provided us with these statistics upon request. We contacted all authors of the studies from which we were unable to extract effect sizes or confidence intervals directly.

### 2.2. Database Search Procedures

The team developed a search strategy focused on the impact of the COVID lockdown on sedentary behavior (see Tables S1–S5, Supplementary Materials which shows the database search strategies) and applied this strategy to Ovid MEDLINE ALL <1946 to May 25, <2022 (770 citations), EMBASE (475 citations), CINAHL (1534 citations), PsycINFO (576 citations), and SportDiscus (130 citations) for a total of 3485 citations, which after deduplication, came to 3056 citations. We used the Ovid COVID-19 search, which was adapted from the COVID-19 PubMed filter [23].

### 2.3. Review Procedure

**Figure 1:**
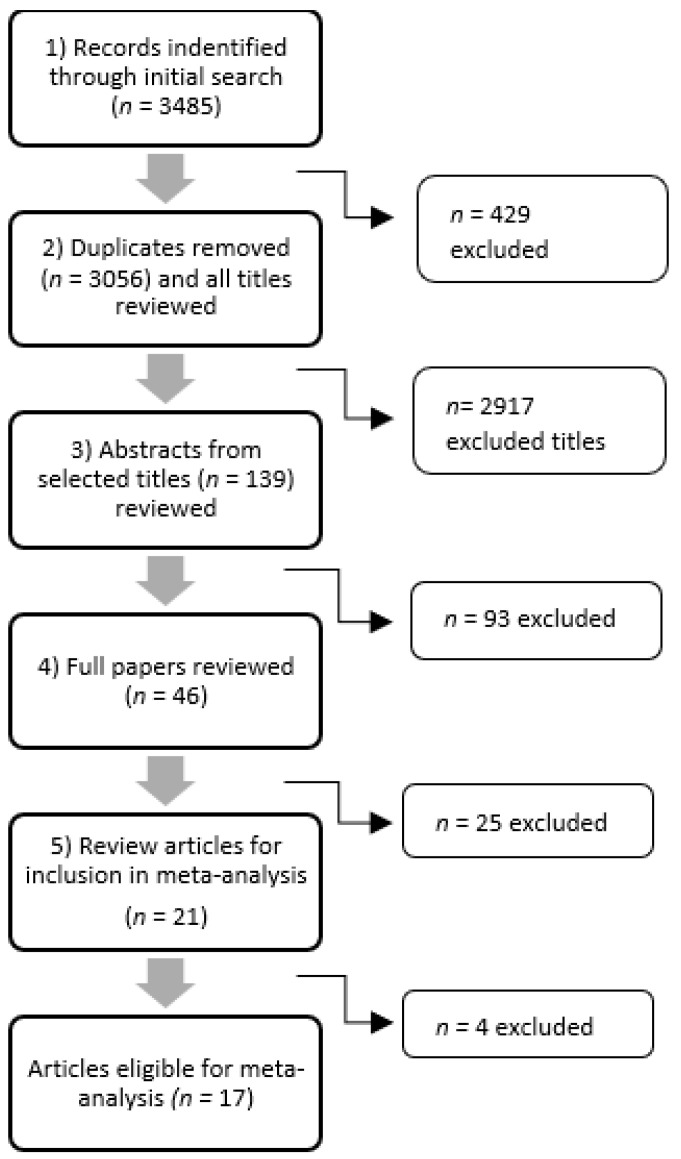
Flowchart representing the methods used to identify the articles for inclusion.

All articles went through a five-step selection process, which is illustrated in Figure 1 and can be summarized as follows: (1) The original search generated 3485 articles. After deduplication, 429 articles were removed, resulting in 3056 articles for review. (2) Two authors (N.C. and M.J.) independently reviewed 3056 titles, and 2917 of these articles did not meet the inclusion criteria, resulting in 139 articles for review. Articles at this stage were primarily excluded due to not being published in English, lack of relevance to the research question, and not being original works (e.g., they were reviews or meta-analyses). (3) Two authors (N.C. and M.J.) read the selected 139 selected abstracts and removed 93 articles, resulting in 46 articles. Articles were primarily excluded due to their data not being collected during the pandemic, not including people working from home as a direct result of the pandemic, or not measuring physical activity or sedentary behavior. (4) Two authors (N.C. and M.J.) read the full 46 selected articles and made final decisions on inclusion, and any disagreements were reconciled by a third author (S.S.T.), which resulted in 21 articles total. Upon a full review of the articles, some articles were excluded primarily due to their data not being collected during the pandemic, not including people working from home as a direct result of the pandemic, or not measuring physical activity or sedentary behavior. Additional studies were excluded because they had samples where a majority of the participants were students or they did not separate working populations from the non-working populations in the analyses. (5) Finally, inclusion for the meta-analysis was assessed by a fourth author (S.P.M.R., a statistical expert), resulting in 17 articles for inclusion. Articles removed at the final review stage were primarily due to not providing enough information to estimate the correct effects or standard errors even after study authors were contacted. After the final articles were identified, data extraction and a bias assessment were performed by 2 authors (M.J and S.S.T).

### 2.4. Data Extraction for the Meta-Analysis

The following were extracted for the meta-analysis: (1) study, (2) study type, (3) outcome, (4) comparison, (5) effect size (Hedge’s *g*), and (6) standard error. The overall effect (Hedge’s *g*) [95% CI], Cochran’s Q, tau, and dispersion of effect sizes (I^2^) were calculated using a random effects model. In cases where data were not presented in Hedge’s *g* format (e.g., odds ratios and correlations), we converted the effect sizes to Hedge’s *g* [24].

## 3. Results

Twenty-one studies were identified, and seventeen studies were included in the meta-analysis. The studies were conducted in the United States [2,5,8,25,26], Brazil [27], Japan [3,9,21,28,29], Canada [26], Poland [30], Italy [10], the Netherlands [7], Sweden [4], France, and Estonia [20], or they were multi-national [6]. One study [31] did not include changes in physical activity before or after the transition or pre-lockdown and during the COVID–19 lockdown; hence, it could not be combined with other studies that looked at such changes. Furthermore, three additional papers did not provide enough information to estimate the correct effects or standard errors and were also excluded from the meta-analysis [31,32,33,34].

Ultimately, 15 studies were included for physical activity comparisons (see Table 1), with a total sample size of 36,650. We included 12 studies for sedentary behavior comparisons (see Table 2), with a total sample size of 57,357. Ten of these studies provided data for both physical activity and sedentary behavior. There were significant decreases in workers’ physical activity levels due to COVID-related lockdowns or remote work implementations (overall effect of −0.29 [−0.41, −0.18]). There were also significant increases in workers’ sedentary behaviors due to COVID-related lockdowns or remote work (overall effect of 0.36 [0.20, 0.52]). The effect sizes in both cases were small. Standard meta-analysis tests including Cochrane’s Q test (1954), [35] and Higgins and Thompson’s test (2002) [36] were run to check for heterogeny (see Table 1 and Table 2). These effects did not differ by dependent vs independent group comparisons, data type (objective vs subjective), or study type (cross-sectional vs longitudinal).

Each study varied in terms of study type, comparison, data type, and population. Many of the studies were cross-sectional, either comparing pre-and post-pandemic behavior in a single survey, or comparing individuals working from home to individuals working on site. Additionally, some studies used independent comparisons (between people who WFH vs those who did not WFH during COVID lockdowns) or dependent comparisons (between WFH and non-WFH times within individuals). The studies also varied in terms of how they collected data. A number of studies collected data using subjective outcomes like surveys, while other studies used objective outcomes like accelerometers or step counters to track activity throughout the day. While the demographics of the participants across all studies varied, the work environments largely consisted of offices, which enabled workers to work from home during the pandemic.

### Bias Assessment

To assess bias within this review, the Cochrane Risk Of Bias In Non-randomized Studies of Interventions (ROBINS-I) tool was used [37]. This assessment has the following seven domains: confounding, selection of participants, classification of interventions, deviations from intended interventions, missing data, measurement of the outcome, and selection of reported results, and each included article was assessed using this tool [37]. Each domain was scored on a 1–4 scale, with 1 being low risk of bias, 2 being moderate risk of bias, 3 being serious risk of bias, and 4 being critical risk of bias, with N/A meaning that there was no information (using the associated interpretation of the tool) [37]. The numbers 1 through 4 were assigned to each level and then averaged to determine an overall bias score. Two authors (M.J. and S.S.T.) independently scored each paper on all domains. If the two authors agreed on the score, then the consensus score was used. If they disagreed on the score, then they reconciled the score.

The greatest bias was observed in the confounding domain, which was expected due to none of the studies being randomized controlled trials. Table 3 summarizes the reconciled authors’ findings. Many of the studies were retrospective designs, and this likely increased the recall bias. The measurement of the outcome domain also frequently scored higher due to most of the data coming from self-reported questionnaires. Even though many of these questionnaires were validated, there was still a risk of recall and reporting bias. Additionally, we ran a moderation test using low or moderate bias as subgroups and found no significant difference in the effect for either physical activity (*p* = 0.53) or sedentary behavior (*p* = 0.32). Overall, the levels of bias detected in the included studies did not warrant concern about their inclusion in the meta-analysis.

## 4. Discussion

Our meta-analysis revealed that in working adults globally, the transition to working from home due to the COVID-19 pandemic resulted in significant reductions in physical activity levels and significant increases in sedentary behavior. The average effect on both outcomes was mild. In terms of physical activity, only two studies in the sample did not report decreases in physical activity [2,5]. One study found that essential employees (i.e., those who had to work in-person to fulfill their job duties during the pandemic) had less sedentary behavior and had more occupational activity than non-essential employees (i.e., those whose work could be moved to home during the pandemic) [5]. This was understandable, as essential employees were called to action during the pandemic and would likely have had more PA associated with their work. The other study found that physical activity levels remained stable, and participants indicated positive changes due to the pandemic, such as more time spent performing enjoyable activities and more time outdoors [2]. These effects may have been unique to the time associated with the pandemic and should be further explored to determine if these results held or changed with society lifting pandemic-related restrictions.

Other studies have shown both positive and negative differences in stress, job satisfaction, and turnover between those who voluntarily chose to work from home compared to those who were forced to work from home [38]. The studies included in this analysis were conducted at a time when working from home was not voluntary. Studying the effects of working from home on PA and SB while offering choices to workers may have affected the working experience and could affect the consistency of this funding in the future. Future research should examine PA and SB by job field, job type, and voluntariness of working from home to obtain a clear picture of how each occupation and working environment may be affected by a change to remote work. Working from home has persisted beyond the pandemic, and daily PA and SB may still be affected by changes in the working environments [39].

Work-related activities, such as commuting, can also greatly affect PA and SB. Previous work has found active commuting, such as biking or walking, to be associated with a lower risk of mortality via cardiovascular risk or cancer compared to passive commuting (i.e., driving) [40]. At the same time, long commutes can increase the risk factors relating to cardiovascular disease [41]. However, active commuting may not be available to those who work from home or certain in-person workers due to commute preferences, available time, transportation infrastructure, or proximity to work. The effect of commuting to work and its effects on PA and SB needs to be evaluated independently from occupational PA and SB.

Furthermore, the change to working from home uniquely affected each person as they faced different home and work environments. The interactions between the home and work environments, individual preferences, and personal experiences working from home would likely contribute to the desire to work from home. Indeed, there is evidence that shows that those who intended to work from home before the pandemic were more likely to work from home during or after the pandemic [42]. Several factors affecting the home-work dynamic, such as individual personality, gender, boundary preference, commute, training, social support, mental well-being, work/family balance, and ergonomic or technology support, also affected the work-from-home experience [38]. Individual preferences and experiences may drive an individual to seek out office or home jobs. After the pandemic, many workplaces shifted to remote work models or hybrid (part WFH and part in-office models). It is important to determine which occupations continue to be affected by workplace changes and how they may affect working adults. This review covered the first 30 months of the pandemic and included the period of time when restrictions began to lift. In this case, we still saw decreases in PA and increases in SB as far as 2 years beyond the start of the pandemic. Future work should consider occupation type and a control for some of the above preferences when examining the effects on PA and SB.

### 4.1. Physical Activity and Sedentary Behavior Counterbalance

It is well-documented that physical activity is beneficial for overall health; as a result, the CDC has clear guidelines for participation in physical activity to maintain overall health [11]. It is also recommended by the World Health Organization (WHO) that adults should reduce their sedentary time due to its association with poor health outcomes [12]. These behaviors can collectively and independently influence health. After adjusting for several confounding factors, including physical activity, prolonged sitting has been found to be a risk factor for all-cause mortality [43]. Furthermore, people who sit for 11 or more hours a day but exercise for ≥300 min a day may have a similar mortality rate to people who also sit for 11 or more hours a day and only engage in 1–149 min of physical activity, indicating that exercise may not be able to reverse the effects of extended sedentary times [43]. Physical activity and sedentary behavior exist as a counterbalance, and as physical activity increases, sedentary behavior is likely to decrease. Therefore, occupational interventions that seek to decrease sedentary time and increase physical activity would likely be a great benefit to workers. The literature shows that breaking up a 3 h sitting segment with 5 min of light physical activity protects cardiovascular function, which could easily be implemented in a work-from-home environment [44]. Workplace supports to increase PA and decrease SB include treadmill desks [45], sit-and-stand stations, and pedal machines [46]. In a recent review on teleworking and worker well-being, workers who reported more workplace flexibility also reported more physical activity, indicating that when workers had more control over their daily schedules, they could make time for PA [38]. Similarly, working from home presents the opportunity to replace a passive commute (e.g., driving) with physical activity, which could be beneficial to worker health [38]. Overall, the emerging evidence on teleworking indicates positive changes in employee sleep, physical activity, and nutrition [38]. In translating our results to public health, a persistent combination of increased sedentary behavior and decreased physical activity in workers could contribute to negative health outcomes. An additional review on the effectiveness of workplace wellness programs based on physical activity showed that programs that included nutritional components; education/counseling regarding health, stress, and nutrition; adaptive physical activity training; financial incentives; environmental interventions; and behavior change interventions were effective in increasing physical activity among workers [47]. This work offers a range of interventions for policy makers and employers to adapt workplace policies and interventions tailored to their workforces to help improve occupational physical activity. Furthermore, as the nature of work for many people across the globe has changed, these health behaviors may continue to persist beyond the pandemic era.

### 4.2. Strengths and Limitations

It is important to consider that these data were collected during the pandemic, which is a less-than-stable environment. For instance, other studies have found that sudden changes in the nature of work could lead to a lack of job control/job loss and may affect activity patterns [48]. Employers may not have been well-prepared for a smooth transition to working from home. Additionally, it is possible that physical activity and sedentary behavior in workers could have been affected by the presence of COVID-19 illnesses in individuals or families. Lastly, there may have been differences across the world in the nature of the lockdowns, consideration of essential vs non-essential services, and cultural and domestic roles of workers [49]. Despite these considerations, this analysis included studies through a 30-month period following the start of the pandemic, which provided a look at the initial phase of the pandemic and continued into the period where restrictions began to lift so that we could see how PA and SB were altered due to the pandemic and throughout the pandemic.

Since most studies used self-report measures for physical activity and sedentary behavior, there may have been a social desirability bias, meaning that the participants tended to over-report physical activity and under-report sedentary behavior [50]. Nonetheless, we found significant decreases in physical activity and significant increases in sedentary behavior, and so we speculated that the strength of our findings may have been enhanced by social desirability bias, thus making the true effect potentially larger. Future work may consider using activity monitors to obtain more accurate pictures of PA and SB in workers. Nevertheless, our overall results demonstrated that workers were more sedentary and less physically active due to working from home as a result of the COVID-19 pandemic, and this may have increased working adults’ risks for chronic illnesses such as cardiovascular disease, obesity, and diabetes [11].

Lastly, although the number of papers used for the meta-analysis was small (*n* = 17), the sample sizes for physical activity (*n* = 36,650) and sedentary behavior (*n* = 57,254) likely represented a snapshot of how PA and SB were altered due to work during the pandemic. However, the studies that were included mostly used a cross-sectional design, suggesting a lack of causal inference. Due to the evolving nature of the pandemic, the papers cited within this meta-analysis can form an important foundation for future longitudinal studies. Our review provides an overview of these critical behaviors that are influenced by rapidly changing work environments. We also included studies that collected data throughout the pandemic, as mandates and lockdowns were changing. Furthermore, these studies included different forms of data collection (i.e., subjective vs objective), and there was a lack of clinically relevant data (i.e., measured minutes of physical activity). Despite these limitations, a strength of our study was that our results have high external validity due to the large sample size and inclusion of multiple national studies.

### 4.3. Future Directions

Whether these results held during the later periods of the pandemic (May 2022 onwards) requires further evaluation. Future studies that evaluate and compare the associations between physical activity and sedentary behavior with health outcomes, between remote workers and on-site workers, or between job types (remote vs on-site) could be useful. Moreover, standardization of the reporting effects in clinically relevant units (e.g., minutes per day/week) would help future meta-analyses provide more clinically relevant information, and this is highly encouraged. As working from home becomes a more common practice, a Total Worker Health^®^ approach, a model where worker health and well-being are advanced through occupational safety and health promotion, may be best utilized to analyze and protect worker health [51].

## 5. Conclusions

The transition to working from home during the first two and a half years of the COVID-19 pandemic significantly reduced physical activity and increased sedentary behavior in working adults globally. The COVID-19 pandemic significantly altered how society functions, and some of the changes persist several years after the pandemic, including how people work. If these results continue to hold, it could significantly impact workers’ long-term health. Our results inform the National Institute for Occupational Safety and Health’s (NIOSH) Future of Work Initiative [52] by presenting findings from which new research and interventions could built upon to protect worker health. Our results can inform multiple stakeholders, including working adults, employers, policymakers, human resources personnel, researchers, and occupational health and safety workers, who can improve the future of work by promoting worker health and advancing further research.

## Figures and Tables

**Table 1:** The physical activity meta-analysis results.

Study Characteristics							Meta-Analysis Results			
Study	Sample Size	Type	Data Type	Comparison	Hedge’s *g*	SE	Overall Effect [95% CI]	*Q*	Tau^2^	*I* ^2^
Ammar et al. (2020) [6]	1047	Cross-sectional	Subjective	Dependent	−0.45	0.03	−0.29 * [−0.41, −0.18]	410.69 *	0.05	96.6%
Argus and Pääsuke (2021) [14]	161	Cross-sectional	Subjective	Dependent	−0.26	0.07				
Azuma et al. (2021) [15]	1086	Retrospective observational	Objective	Independent	−0.23	0.06				
Barone Gibbs et al. (2021) [2]	112	Prospective	Subjective	Dependent	0.18 ^a^	0.17				
Fukushima et al. (2021) [3]	1239	Cross-sectional	Subjective	Independent	−0.45 ^b^	0.06				
Guler et al. (2021) [18]	194	Cross-sectional	Subjective	Dependent	−0.13	0.05				
Hallman et al. (2021) [4]	27	Prospective	Objective	Dependent	−0.04 ^c^	0.23				
Howe et al. (2021) [19]	687	Cross-sectional	Subjective	Dependent	−0.46	0.06				
Lindsey et al. (2021) [5]	737	Cross-sectional	Subjective	Independent	0.16 ^d^	0.09				
Lipert et al. (2021) [23]	1959	Cross-sectional	Subjective	Independent	−0.45	0.07				
Loef et al. (2022) [7]	18,379	Longitudinal	Subjective	Independent	−0.05	0.01				
McDowell et al. (2020) [8]	2454	Cross-sectional	Subjective	Independent	−0.03	0.04				
Nagata et al. (2021) [9]	896	Longitudinal	Objective	Independent	−0.37	0.07				
Rapisarda et al. (2021) [10]	310	Longitudinal	Subjective	Dependent	−0.72	0.06				
Yoshimoto et al. (2021) [22]	1941	Cross-sectional	Subjective	Independent	−0.47	0.06				

Note: SE is the standard error of the study. The overall effect is the weighted g from the random effects model including the 95% confidence interval. Q is the Cochrane’s Q test (1954) for heterogeneity. The tau^2^ values were computed with the Paule–Mendel estimator. *I*^2^ is the Higgins and Thompson (2002)’s test of heterogeneity. ^a^ denotes the medians and interquartile ranges that were reported in the original papers. Means and standard deviations were used to compute the effect sizes that were provided by the authors. ^b^ denotes the effect sizes representing the average g values for light physical activity and moderate-to-vigorous physical activity. ^c^ denotes the effect sizes representing the average g values for physical activity at work and physical activity during leisure time. ^d^ denotes the effect sizes representing the average g values for light physical activity, moderate physical activity, and vigorous physical activity. * denotes statistical significance, with *p* < 0.05.

**Table 2:** Sedentary behavior meta-analysis results.

Study Characteristics							Meta-Analysis Results			
Study	Sample Size	Type	Outcome	Comparison	Hedge’s *g*	SE	Overall Effect [95% CI]	*Q*	Tau^2^	*I* ^2^
Ammar et al. (2020) [6]	1047	Cross-sectional	Subjective	Dependent	0.67	0.03	0.36 * [0.20, 0.52]	864.08 *	0.07	98.7%
Barone Gibbs et al. (2021) [2]	112	Prospective	Subjective	Dependent	0.19	0.11				
Fukushima et al. (2021) [3]	1239	Cross-sectional	Subjective	Independent	0.67	0.06				
Hallman et al. (2021) [4]	27	Prospective	Objective	Dependent	−0.11 ^a^	0.22				
Koyama et al. (2021) [21]	11,623	Cross-sectional	Subjective	Independent	0.42	0.05				
Lindsey et al. (2021) [5]	737	Cross-sectional	Subjective	Independent	0.28	0.09				
Loef et al. (2022) [7]	18,379	Longitudinal	Subjective	Independent	0.23	0.02				
McDowell et al. (2020) [8]	2454	Cross-sectional	Subjective	Independent	0.15	0.03				
Nagata et al. (2021) [9]	896	Cross-sectional	Subjective	Independent	0.44	0.09				
Rapisarda et al. (2021) [10]	310	Longitudinal	Subjective	Dependent	0.86	0.03				
Silva et al. (2021) [20]	39,693	Cross-sectional	Subjective	Independent	−0.03	0.02				

Note. SE is the standard error of the study. The overall effect is the weighted g from the random effects model including the 95% confidence interval. Q is the Cochrane’s Q test (1954) for heterogeneity. The tau^2^ values were computed with the Paule–Mendel estimator. *I*^2^ denotes the Higgins and Thompson (2002) test of heterogeneity. ^a^ denotes the effect size representing the average g values for sedentary behavior at work and during leisure time. * denotes statistical significance, with *p* < 0.05.

**Table 3:** Final bias assessment values.

Bias Domains								
Paper	Confounding	Selection of Participants	Classification of Interventions	Deviations from Intended Interventions	Missing Data	Measurement of the Outcome	Selection of Reported Result	Overall Bias
Ammar et al. (2020) [6]	2	2	1	N/A	1	1	1	1.33
Argus and Pääsuke (2021) [20]	2	1	2	N/A	1	2	1	1.5
Azuma et al. (2021) [21]	2	2	1	N/A	1	2	1	1.5
Barone Gibbs et al. (2021) [2]	2	2	1	N/A	1	1	1	1.33
Fukushima et al. (2021) [3]	2	2	2	N/A	1	1	1	1.5
Guler et al. (2021) [25]	2	1	1	N/A	1	2	1	1.33
Hallman et al. (2021) [4]	2	2	1	N/A	2	1	1	1.5
Howe et al. (2021) [19]	2	2	1	N/A	1	2	1	1.5
Koyama et al. (2021) [21]	2	2	2	N/A	1	2	2	1.66
Lindsey et al. (2021) [5]	2	1	1	N/A	1	2	1	1.33
Lipert et al. (2021) [30]	2	2	1	N/A	1	2	1	1.5
Loef et al. (2022) [7]	2	1	1	N/A	1	1	1	1.16
McDowell et al. (2020) [8]	2	2	1	N/A	1	2	2	1.66
Nagata et al. (2021) [9]	2	2	1	N/A	1	2	1	1.5
Rapisarda et al. (2021) [10]	1	1	1	N/A	1	2	1	1.16
Silva et al. (2021) [27]	2	2	1	N/A	1	2	1	1.5
Yoshimoto et al. (2021) [29]	2	2	1	N/A	1	2	1	1.5

Each domain was scored on a 1–4 scale, with 1 being low risk of bias, 2 being a moderate risk of bias, 3 being a serious risk of bias, and 4 being a critical risk of bias, with N/A referring to no information, using the Cochrane Risk Of Bias In Non-randomized Studies of Interventions (ROBINS-I) tool.

## Data Availability

No new data were created or analyzed in this study.

## Supplementary Materials

The following supporting information can be downloaded at: https://www.mdpi.com/article/10.3390/ijerph21070851/s1, Table S1: OVID search strategy; Table S2: EMBASE search strategy; Table S3: CINAHL plus with full text (cumulative index to nursing and allied health literature) through the EBSCOHost search strategy; Table S4: PSYCINFO through the Ovid search strategy; and Table S5: SPORTDISCUS through the EbscoHost search strategy. The SportDiscus search was adapted from the Ovid search.
