# Supplementary material for: Transitioning to Working from Home Due to the COVID-19 Pandemic Significantly Increased Sedentary Behavior and Decreased Physical Activity: A Meta-Analysis
Source: Int J Environ Res Public Health. 2024 Jun 28;21(7):851. doi: 10.3390/ijerph21070851 (PMC11276674; doi:10.3390/ijerph21070851)
Supplement: Supplementary file 1 [file ijerph-21-00851-s001.zip › ijerph-3013297-supplementary.pdf]

International Journal of
Environmental Research and Public Health

MDPI

SUPPLEMENTARY MATERIALS

Table S1. OVID search strategy.

	**Database: Ovid MEDLINE(R) ALL <1946 to May 25, 2022>**
1	(telework* or tele-work* or telecommut* or tele-commut* or cybercommut* or cyberwork* or cyber-work* or cyber-commut* or lockdown* or (lock* adj2 down*) or (home* adj5 (remot* or distanc*)) or ((work* or job* or office*) adj5 (remot* or distanc* or home*))).mp. (37758)
2	limit 1 to covid-19 (16303)
3	((forc* or clos* or involuntar* or mandat* or requir* or restrict* or necessar* or home* or lockdown* or (lock* adj2 down*)) adj5 (offic* or workplac*)).mp. [mp=title, abstract, original title, name of substance word, subject heading word, floating sub-heading word, keyword heading word, organism supplementary concept word, protocol supplementary concept word, rare disease supplementary concept word, unique identifier, synonyms] (7840)
4	limit 3 to covid-19 (536)
5	exp Sedentary Behavior/ (12616)
6	motor activity/ or exp exercise/ (321585)
7	exp Physical Fitness/ (34872)
8	exp Musculoskeletal Physiological Phenomena/ (1120400)
9	exp Health Behavior/ (351855)
10	5 or 6 or 7 or 8 (1124884)
11	1 and 10 (1662)
12	2 and 10 (476)
13	(sedentar* or (excess* adj3 (sit or sits or sitting)) or exercis* or (physical* adj3 (fit* or activ* or inactiv* or stamin* or endur* or strength* or strong* or weak* or capac*))).mp. [mp=title, abstract, original title, name of substance word, subject heading word, floating sub-heading word, keyword heading word, organism supplementary concept word, protocol supplementary concept word, rare disease supplementary concept word, unique identifier, synonyms] (550289)
14	1 and 13 (2769)
15	limit 14 to yr="2020 -Current" (1651)
16	1 and 9 (1417)
17	limit 16 to yr="2020 -Current" (409)
18	((sedentar* or (excess* adj3 (sit or sits or sitting)) or exercis* or (physical* adj3 (fit* or activ* or inactiv* or stamin* or endur* or strength* or strong* or weak* or capac*))) adj12 (telework* or tele-work* or telecommut* or tele-commut* or cybercommut* or cyberwork* or cyber-work* or cyber-commut* or (home* adj5 (remot* or distanc*)) or ((work* or job* or office*) adj5 (remot* or distanc* or home*)))).mp. [mp=title, abstract, original title, name of substance word, subject heading word, floating sub-heading word, keyword heading word, organism supplementary concept word, protocol supplementary concept word, rare disease supplementary concept word, unique identifier, synonyms] (569)
19	limit 18 to yr="2020 -Current" (190)
20	exp work/ (68743)
21	exp occupational groups/ (684767)
22	exp Psychology, Industrial/ (93487)
23	exp Workforce/ (79321)
24	exp Personnel Management/ (206670)
25	21 or 22 or 23 or 24 (907432)
26	2 and 25 (1081)
27	10 and 26 (29)
28	13 and 26 (54)
29	27 or 28 (58)
30	4 and 25 (85)
31	10 or 13 (1390347)
32	30 and 31 (6)
33	27 or 28 or 29 or 30 or 32 (138)
34	10 and 25 (25750)
35	limit 34 to covid-19 (90)
36	17 or 19 or 33 or 35 (770)

Table S2. EMBASE search strategy.

((((('sedentary lifestyle'/exp OR 'sitting'/exp OR 'sedentary time'/exp) OR ('exercise'/exp OR 'motor activity'/exp OR 'physical activity'/exp OR 'fitness'/exp OR 'musculoskeletal function'/exp)) AND ((('coronavirus disease 2019'/exp OR 'severe acute respiratory syndrome coronavirus 2'/exp OR ('covid-19' OR covid19 OR ((covid* OR coronavir*) NEAR/3 pandemic*))) AND (telework* OR 'tele work*' OR telecommut* OR 'tele commut*' OR cybercommut* OR cyberwork* OR 'cyber work*' OR 'cyber commut*' OR lockdown* OR (lock* NEAR/2 down*) OR (home* NEAR/5 (remot* OR distanc*)) OR ((work* OR job* OR office*) NEAR/5 (remot* OR distanc* OR home*)))) AND ('work'/exp OR 'named groups by occupation'/exp OR 'occupational psychology'/exp OR 'workforce'/exp OR 'personnel management'/exp))) OR (((sedentar* OR exercis*) NEAR/10 (work* OR job* OR office*) AND (work* OR job* OR office*) NEAR/5 (remot* OR distanc* OR home*)) AND ((('coronavirus disease 2019'/exp OR 'severe acute respiratory syndrome coronavirus 2'/exp OR ('covid-19' OR covid19 OR ((covid* OR coronavir*) NEAR/3 pandemic*))) AND (telework* OR 'tele work*' OR telecommut* OR 'tele commut*' OR cybercommut* OR cyberwork* OR 'cyber work*' OR 'cyber commut*' OR lockdown* OR (lock* NEAR/2 down*) OR (home* NEAR/5 (remot* OR distanc*)) OR ((work* OR job* OR office*) NEAR/5 (remot* OR distanc* OR home*)))) AND ('work'/exp OR 'named groups by occupation'/exp OR 'occupational psychology'/exp OR 'workforce'/exp OR 'personnel management'/exp))) OR (('coronavirus disease 2019'/exp OR 'severe acute respiratory syndrome coronavirus 2'/exp OR ('covid-19' OR covid19 OR ((covid* OR coronavir*) NEAR/3 pandemic*))) AND (forc* OR clos* OR involuntar* OR mandat* OR requir* OR restrict* OR necessar* OR home* OR lockdown* OR 'lock down' OR 'locked down' OR 'locking down') NEAR/5 (offic* OR workplac*) AND (('sedentary lifestyle'/exp OR 'sitting'/exp OR 'sedentary time'/exp) OR ('exercise'/exp OR 'motor activity'/exp OR 'physical activity'/exp OR 'fitness'/exp OR 'musculoskeletal function'/exp)) AND ('work'/exp OR 'named groups by occupation'/exp OR 'occupational psychology'/exp OR 'workforce'/exp OR 'personnel management'/exp)) OR (('covid-19' OR covid19 OR ((covid* OR coronavir*) NEAR/3 pandemic*)) AND ((sedentar* OR exercis*) NEAR/10 (work* OR job* OR office*) AND (work* OR job* OR office*) NEAR/5 (remot* OR distanc* OR home*)))) AND [embase]/lim) NOT ((((('sedentary lifestyle'/exp OR 'sitting'/exp OR 'sedentary time'/exp) OR ('exercise'/exp OR 'motor activity'/exp OR 'physical activity'/exp OR 'fitness'/exp OR 'musculoskeletal function'/exp)) AND ((('coronavirus disease 2019'/exp OR 'severe acute respiratory syndrome coronavirus 2'/exp OR ('covid-19' OR covid19 OR ((covid* OR coronavir*) NEAR/3 pandemic*))) AND (telework* OR 'tele work*' OR telecommut* OR 'tele commut*' OR cybercommut* OR cyberwork* OR 'cyber work*' OR 'cyber commut*' OR lockdown* OR (lock* NEAR/2 down*) OR (home* NEAR/5 (remot* OR distanc*)) OR ((work* OR job* OR office*) NEAR/5 (remot* OR distanc* OR home*)))) AND ('work'/exp OR 'named groups by occupation'/exp OR 'occupational psychology'/exp OR 'workforce'/exp OR 'personnel management'/exp))) OR (((sedentar* OR exercis*) NEAR/10 (work* OR job* OR office*) AND (work* OR job* OR office*) NEAR/5 (remot* OR distanc* OR home*)) AND ((('coronavirus disease 2019'/exp OR 'severe acute respiratory syndrome coronavirus 2'/exp OR ('covid-19' OR covid19 OR ((covid* OR coronavir*) NEAR/3 pandemic*))) AND (telework* OR 'tele work*' OR telecommut* OR 'tele commut*' OR cybercommut* OR cyberwork* OR 'cyber work*' OR 'cyber commut*' OR lockdown* OR (lock* NEAR/2 down*) OR (home* NEAR/5 (remot* OR distanc*)) OR ((work* OR job* OR office*) NEAR/5 (remot* OR distanc* OR home*)))) AND ('work'/exp OR 'named groups by occupation'/exp OR 'occupational psychology'/exp OR 'workforce'/exp OR 'personnel management'/exp))) OR (('coronavirus disease 2019'/exp OR 'severe acute respiratory syndrome coronavirus 2'/exp OR ('covid-19' OR covid19 OR ((covid* OR coronavir*) NEAR/3 pandemic*))) AND (forc* OR clos* OR involuntar* OR mandat* OR requir* OR restrict* OR necessar* OR home* OR lockdown* OR 'lock down' OR 'locked down' OR 'locking down') NEAR/5 (offic* OR workplac*) AND (('sedentary lifestyle'/exp OR 'sitting'/exp OR 'sedentary time'/exp) OR ('exercise'/exp OR 'motor activity'/exp OR 'physical activity'/exp OR 'fitness'/exp OR 'musculoskeletal function'/exp)) AND ('work'/exp OR 'named groups by occupation'/exp OR 'occupational psychology'/exp OR 'workforce'/exp OR 'personnel management'/exp)) OR (('covid-19' OR covid19 OR ((covid* OR coronavir*) NEAR/3 pandemic*)) AND ((sedentar* OR exercis*) NEAR/10 (work* OR job* OR office*) AND (work* OR job* OR office*) NEAR/5 (remot* OR distanc* OR home*)))) AND [medline]/lim) AND [2020-2022]/py

----

Table S3. CINAHL plus with full text (cumulative index to nursing and allied health literature) through the EBSCOHost search strategy.

#	Query	Results
S41	S38 OR S40	1,534
S40	S1 AND S11	409
S39	S1 AND S11	1,611
S38	S33 OR S35 OR S37	1,168
S37	S2 AND S36	44
S36	((sedentar* or (excess* N3 (sit or sits or sitting)) or exercis* or (physical* N3 (fit* or activ* or inactiv* or stamin* or endur* or strength* or strong* or weak* or capac*))) N12 (telework* or tele-work* or telecommut* or tele-commut* or cybercommut* or cyberwork* or cyber-work* or cyber-commut* or (home* N5 (remot* or distanc*)) or ((work* or job* or office*) N5 (remot* or distanc* or home*))))	339
S35	S2 AND S34	670
S34	S12 AND S25	8,480
S33	S27 OR S28 OR S29 OR S30 OR S32	1,135
S32	S30 AND S31	33
S31	S12 OR S15	594,325
S30	S5 AND S25	426
S29	S27 OR S28	742
S28	S15 AND S26	367
S27	S12 AND S26	670
S26	S2 AND S25	17,947
S25	S20 OR S21 OR S22 OR S23 OR S24	121,614
S24	(MH "Personnel Management+") OR "personnel management"	31,667
S23	(MH "Workforce") OR "workforce"	8,591
S22	(MH "Psychology, Occupational+") OR "industrial psychology"	17,280
S21	(MH "Named Groups by Occupation+") OR "occupational groups"	92,813
S20	(MH "Work+")	1,693
S19	S1 AND S11	409
S18	S1 AND S11	1,611
S17	S1 AND S15	1,277
S16	S1 AND S15	4,106
S15	(sedentar* or (excess* adj3 (sit or sits or sitting)) or exercis* or (physical* N3 (fit* or activ* or inactiv* or stamin* or endur* or strength* or strong* or weak* or capac*)))	292,856
S14	S3 AND S12	698
S13	S1 AND S12	5,680
S12	S6 OR S7 OR S8 OR S9 OR S10 OR S11	560,320
S11	(MH "Health Behavior+") OR "health behavior"	118,439
S10	"Musculoskeletal Physiological Phenomena" OR (MH "Musculoskeletal System Physiology+")	339,038
S9	(MH "Physical Fitness+") OR "physical fitness"	22,530
S8	(MH "Exercise+") OR "exercise"	228,610
S7	(MH "Motor Activity+") OR "motor activity" OR (MH "Physical Activity")	62,256
S6	(MH "Life Style, Sedentary+") OR "sedentary"	19,330
S5	S2 AND S4	1,291
S4	((forc* or clos* or involuntar* or mandat* or requir* or restrict* or necessar* or home* or lockdown* or (lock* adj2 down*)) N5 (offic* or workplac*))	25,542
S3	S1 AND S2	7,741
S2	(MH "COVID-19+") OR "covid-19" OR (MH "COVID-19 Pandemic")	103,293
S1	telework OR tele-work OR telecommut* OR tele-commut* OR cybercommut* OR cyberwork* OR cyber-work* or cyber-commut* or lockdown* or (lock* N2 down*) or (home* N5 (remot* or distanc*)) or ((work* or job* or office*) N5 (remot* or distanc* or home*)))	

Table S4. PSYCINFO through the Ovid search stategy.

#	Query	Results
1	(telework* or tele-work* or telecommut* or tele-commut* or cybercommut* or cyberwork* or cyber-work* or cyber-commut* or lockdown* or (lock* adj2 down*) or (home* adj5 (remot* or distanc*)) or ((work* or job* or office*) adj5 (remot* or distanc* or home*))).mp.	18161
2	covid-19.mp. or exp COVID-19/	21098
3	1 and 2	3455
4	((forc* or clos* or involuntar* or mandat* or requir* or restrict* or necessar* or home* or lockdown* or (lock* adj2 down*)) adj5 (offic* or workplac*)).mp.	4464
5	2 and 4	110
6	exp Sedentary Behavior/ or Sedentary Behavior.mp.	3218
7	motor activity.mp.	39273
8	exp Exercise/ or exercise.mp.	74397
9	physical fitness.mp. or exp Physical Fitness/	8216
10	Musculoskeletal Physiological Phenomena.mp.	111
11	health behavior.mp. or exp Health Behavior/	54671
12	7 or 8 or 9 or 10 or 11	160268
13	1 and 12	951
14	5 and 12	19
15	(sedentar* or (excess* adj3 (sit or sits or sitting)) or exercis* or (physical* adj3 (fit* or activ* or inactiv* or stamin* or endur* or strength* or strong* or weak* or capac*))).mp.	129063
16	1 and 15	840
17	limit 16 to yr="2020 -Current"	324
18	1 and 11	552
19	limit 18 to yr="2020 -Current"	345
20	((sedentar* or (excess* adj3 (sit or sits or sitting)) or exercis* or (physical* adj3 (fit* or activ* or inactiv* or stamin* or endur* or strength* or strong* or weak* or capac*))) adj12 (telework* or tele-work* or telecommut* or tele-commut* or cybercommut* or cyberwork* or cyber-work* or cyber-commut* or (home* adj5 (remot* or distanc*)) or ((work* or job* or office*) adj5 (remot* or distanc* or home*))))	191
21	limit 20 to yr="2020 -Current"	38
22	work.mp.	562606
23	exp Occupations/ or occupational groups.mp.	65723
24	industrial psychology.mp. or exp "Industrial and Organizational Psychology"/	8755
25	workforce.mp.	18581
26	Personnel Management.mp. or exp Human Resource Management/	58381
27	22 or 23 or 24 or 25 or 26	647967
28	3 and 27	957
29	12 and 28	88
30	15 and 28	46
31	29 or 30	109
32	5 and 27	49
33	12 or 15	205579
34	32 and 33	8
35	29 or 30 or 31 or 32 or 34	150
36	12 and 27	15841
37	2 and 36	211
38	19 or 21 or 35 or 37	576
39	limit 38 to yr="2020 -Current"	576

Table S5. SPORTDISCUS through the EbscoHost search strategy. The SportDiscus search was adapted from the Ovid search.

#	Query	Results
S40	S30 OR S31 OR S32 OR S33 OR S34 OR S35 OR S37 OR S38 OR S39	130
S39	S5 AND S36	3
S38	S1 AND S7	70
S37	S2 AND S36	30
S36	((sedentar* or (excess* N3 (sit or sits or sitting)) or exercis* or (physical* N3 (fit* or activ* or inactiv* or stamin* or endur* or strength* or strong* or weak* or capac*))) N12 (telework* or tele-work* or telecommut* or tele-commut* or cybercommut* or cyberwork* or cyber-work* or cyber-commut* or (home* N5 (remot* or distanc*)) or ((work* or job* or office*) N5 (remot* or distanc* or home*))))	238
S35	S30 OR S31 OR S32 OR S33 OR S34	36
S34	S23 AND S33	1
S33	S5 AND S16	5
S32	S30 OR S31	32
S31	S11 AND S29	28
S30	S8 AND S29	24
S29	S2 AND S28	185
S28	SU work OR SU occupational groups OR SU industrial psychology OR SU occupational psychology OR SU workforce OR SU personnel management	1,610
S27	S1 AND S7	38
S26	S1 AND S7	70
S25	S1 AND S11	327
S24	S1 AND S11	982
S23	S6 OR S11	365,260
S22	S5 AND S11	8
S21	S5 AND S6	6
S20	S18 OR S19	6
S19	S11 AND S17	5
S18	S8 AND S17	6
S17	S3 AND S16	25
S16	SU work OR SU occupational groups OR SU industrial psychology OR SU occupational psychology OR SU workforce OR SU personnel management	1,610
S15	S1 AND S7	38
S14	S1 AND S7	70
S13	S1 AND S11	327
S12	S1 AND S11	982
S11	(sedentar* or (excess* adj3 (sit or sits or sitting)) or exercis* or (physical* N3 (fit* or activ* or inactiv* or stamin* or endur* or strength* or strong* or weak* or capac*)))	365,260
S10	S4 AND S8	107
S9	S1 AND S8	731
S8	S6 OR S7	239,937
S7	SU health behavior*	9,685
S6	SU sedentary OR SU motor activity OR SU physical activity OR SU exercise OR SU physical fitness OR SU exercise OR SU Musculoskeletal Physiological Phenomena	234,714
S5	S2 AND S4	44
S4	((forc* or clos* or involuntar* or mandat* or requir* or restrict* or necessar* or home* or lockdown* or (lock* adj2 down*)) N5 (offic* or workplac*))	1,473
S3	S1 AND S2	712
S2	SU covid-19 OR SU covid-19 pandemic OR TX covid-19	13,658
S1	telework OR tele-work OR telecommut* OR tele-commut* OR cybercommut* OR cyberwork* OR cyber-work* or cyber-commut* or lockdown* or (lock* N2 down*) or (home* N5 (remot* or distanc*)) or ((work* or job* or office*) N5 (remot* or distanc* or home*)))	3,558
